# Predictors of postoperative cardiovascular complications up to 3 months after kidney transplantation

**DOI:** 10.1007/s12471-020-01373-6

**Published:** 2020-02-17

**Authors:** W. K. den Dekker, M. C. Slot, M. M. L. Kho, T. W. Galema, J. van de Wetering, E. Boersma, J. I. Roodnat

**Affiliations:** 1 Department of Cardiology, Thoraxcenter, Erasmus Medical Centre, University Medical Centre Rotterdam, Rotterdam, The Netherlands; 2 Department of Allergology, Maastricht University Medical Centre, Maastricht, The Netherlands; 3 Department of Internal Medicine, Erasmus Medical Centre, University Medical Centre Rotterdam, Rotterdam, The Netherlands

**Keywords:** Cardiac screening, Kidney transplantation, Non-invasive ischaemia detection, Coronary revascularisation

## Abstract

**Background:**

Renal transplant patients have a high peri-operative risk for cardiovascular events. Pre-operative screening for cardiac ischaemia might lower this risk, but there are no specific guidelines.

**Methods:**

We conducted a chart review for all renal transplants performed between January 2010 and December 2013. We collected data about patient characteristics, pre-operative cardiac evaluation before referral, diagnostic tests and interventions. Logistic regression analyses were then applied to relate these factors to the composite endpoint of cardiac death, myocardial infarction, coronary revascularisation or admission for heart failure within 3 months after transplantation.

**Results:**

A total of 770 kidney transplants were performed in 751 patients. In 750 cases (97%) a referral to the cardiologist was made. Non-invasive ischaemia detection by myocardial perfusion scintigraphy, exercise stress test or dobutamine stress echocardiography was carried out in 631 cases (82%). Coronary angiography was performed in 85 cases, which revealed significant coronary artery disease in 19 cases. Prophylactic revascularisation was done in 7 cases. The incidence of the study endpoint was 8.6%. In multivariable regression analysis, age at transplantation, pre-transplant myocardial infarction or heart failure, post-operative decrease in haemoglobin and positive non-invasive ischaemia testing were significantly associated with the study endpoint. However, when analysed separately, none of the different non-invasive ischaemia detection modalities were related to the study endpoint.

**Conclusion:**

Especially those renal transplant candidates with a cardiac history carry a high risk for a cardiovascular event post-transplantation. Uniformity in cardiac screening of renal transplant candidates and better pre-operative preparation might lower this post-operative risk. Besides, post-transplant anaemia should be prevented.

## What’s new?

- Kidney transplantation should be considered a high-risk procedure for post-operative cardiac events.
- There are no specific guidelines for pre-operative cardiac screening.
- Uniformity in cardiac screening of renal transplant candidates, especially in ischaemia detection, is warranted.
- Pre-operative revascularisation does not seem to be associated with a better outcome.

## Introduction

Patients with end-stage renal disease (ESRD) have a high risk of cardiovascular events [1] and a low quality of life [2, 3]. Patients with ESRD are often asymptomatic, but the number of patients with significant coronary artery stenosis (defined as stenosis >50%) has been shown to be between 37% and 65%, rendering them at high risk for short- and long-term cardiovascular events or even death [4]. To lower this cardiovascular risk and improve quality of life, kidney transplantation can be performed in patients with ESRD [5]. Despite a reduction in overall long-term mortality with transplantation, an increased short-term post-transplant cardiovascular mortality risk has been documented [6]. For example, there is an increased risk of type I myocardial infarction due to plaque rupture because of changes in shear stress, vasospasm and thrombocyte activation, but also of type II myocardial infarction due to blood loss and subsequent anaemia, tachycardia or hypotension [7]. Cardiac screening and pre-transplant treatment might be helpful to lower the post-operative risk of cardiovascular events [8]. However, there is no consensus on indications or methods for screening.

The European Society of Cardiology has published guidelines on cardiovascular assessment and management for non-cardiac surgery [9]. Kidney transplantation is regarded as intermediate-risk surgery with a relatively high threshold for ischaemia detection. Ischaemia detection is deemed appropriate only in patients with poor functional capacity or anginal complaints, and more than one clinical risk factor. However, patients with renal failure display a poor correlation between signs and symptoms and significant coronary artery disease, and less frequently have anginal complaints in the setting of acute or chronic ischaemia [10–12]. Therefore, clinical presentation of ESRD patients may not be very helpful to distinguish which patients should be screened. To avoid this, screening can be determined by the number of risk factors. Risk factors for cardiovascular disease in patients evaluated for renal transplantation were captured in the 2007 Lisbon Conference Report and include diabetes mellitus (DM), prior cardiovascular disease, >1 year on dialysis, left ventricular hypertrophy (LVH), age >60 years, smoking, hypertension and dyslipidaemia [13]. The presence of three or more risk factors should prompt testing [14, 15]. The majority of renal transplant candidates have at least three of these factors.

Current guidelines do not recommend a specific screening test [9]. As many patients that undergo kidney transplantation in this centre are referred from peripheral hospitals and cardiac screening is performed in the referring hospital, there is considerable variation in screening tests used, depending upon local expertise and experience. The most commonly used tests are dobutamine stress echocardiography (DSE), myocardial perfusion scintigraphy (MPS) or exercise stress testing (EST). It is recommended to first perform a non-invasive cardiac screening test, because of the potential risks of coronary angiography (CAG), including contrast nephropathy, bleeding and cerebrovascular accident. Besides variation in the screening method used, a lack of direct feedback on cardiological complications after renal transplantation might hamper proper evaluation in the referring hospital.

In the current study, we evaluated pre-operative cardiac risk assessment and treatment. Furthermore, we evaluated the incidence of cardiovascular events within 3 months after transplantation and defined predictors for these cardiovascular events.

## Methods

### Study population

This is a retrospective study of all consecutive kidney transplants performed between 1 January 2010 and 31 December 2013 at the Erasmus Medical Centre (EMC), Rotterdam. The kidney transplantation department of the EMC is the largest in the Netherlands, performing about 200 deceased- or living-donor kidney transplantations per year. Patients are referred by the nephrology department of the EMC and from at least six referring hospitals. Patients were excluded from the study if they were <18 years of age or received a combined liver-kidney transplant. All patients were followed up for 3 months or until transplant failure or death, whichever came first. As every transplant performed during the period was studied, a patient could be included more than once. Complete re-evaluation was performed before each new registration as a transplant candidate. Study parameters were recorded for every new transplant in this patient.

### Study parameters

For all patients, all available clinical data were reviewed. Three or more risk factors were defined as a binary variable including: DM, prior cardiovascular disease, prior heart failure, >1 year on dialysis, LVH, age >60 years, smoking, hypertension and dyslipidaemia. History of dialysis and type and duration of dialysis were recorded. Type of donor (living or deceased) and delayed graft function (defined as the need for at least one dialysis treatment in the 1st week after kidney transplantation) were noted.

For all patients, pre-operative screening by the cardiologist was recorded. Furthermore, if performed, the results of ischaemia detection by EST, DSE and/or MPS were documented. In some patients, only CAG was performed; in others, CAG was done to further evaluate the results of non-invasive ischaemia detection.

### Events

The primary endpoint was defined as a composite of cardiac death, myocardial infarction, coronary revascularisation or heart failure necessitating admission within 3 months after transplantation. The time frame of 3 months was chosen as most complications post-transplantation (bleeding, sepsis, rejection, restart haemodialysis) occur within 3 months after transplantation. Cardiac death was defined as death with a clear cardiac cause or death of an unknown cause. Only deaths with a documented non-cardiac cause were classified as non-cardiac death. The fourth universal definition of myocardial infarction was used, i.e. a rise and/or fall of cardiac troponin values with at least one value above the 99th percentile upper reference limit in combination with at least one of the following: (1) symptoms of myocardial ischaemia; (2) new ischaemic ECG changes; (3) development of pathological Q waves; (4) imaging evidence of new loss of viable myocardium or new regional wall motion abnormality in a pattern consistent with an ischaemic aetiology; (5) identification of a coronary thrombus by angiography at autopsy. Pre- and post-operative troponin levels or post-operative ECGs are assessed only upon clinical indication. Coronary revascularisation was defined as percutaneous coronary intervention (PCI) or coronary artery bypass graft (CABG) of any coronary artery. Heart failure necessitating admission was defined as the need to treat heart failure with intravenous diuretic, inotropic agent or vasodilator in combination with symptoms and signs of heart failure. All suspected primary endpoints were adjudicated by a single nephrologist (M.S.) and cardiologist (W.D.), who reached a consensus in discrepant transplant candidates.

### Statistical analyses

Categorical data are presented as numbers with percentages and the differences between the patients with and without study endpoint were evaluated using chi-square tests. Continuous data are presented as mean ± standard deviation and differences between these samples were tested using Student’s *t*-tests. Univariable and multivariable logistic regression analyses were applied to study the relation between a broad range of patient characteristics (see Tab. 1) and the study endpoint. The number of variables (degrees of freedom) in the multivariable model was limited to 7, since there were only 66 patients who reached the study endpoint. Therefore, pragmatically, only variables with a *p-*value <0.05 in univariable analysis entered the multivariable stage, whereas the model reduction method of backward elimination was utilised, again applying the *p*-value <0.05 criterion. For all tests, a *p*-value <0.05 (two-sided) was considered statistically significant. Analyses were performed using SPSS version 23 (IBM, Armonk, NY, USA).

**Table 1:** Patient characteristics

Variable	All (*n* = 770)	No cardiac event (*n* = 704)	Cardiac event (*n* = 66)	*p*^a^
Male gender	493 (64%)	452 (64%)	41 (62%)	NS
Age at transplant (mean ± SD)	54.3 ± 13.9	53.4 ± 14.0	63.0 ± 9.6	<0.001
Age above 60	309 (40%)	264 (38%)	45 (68%)	<0.001
Delayed graft function	174 (23%)	149 (21%)	25 (38%)	0.001
Living donor	532 (69%)	499 (71%)	33 (50%)	0.001
Dialysis before kidney transplantation	522 (68%)	468 (66%)	54 (82%)	0.001
Dialysis duration longer than 1 year	374 (49%)	331 (47%)	43 (65%)	0.004
History of cardiac disease	139 (18%)	100 (14%)	39 (59%)	<0.001
– Intervention	86 (11%)	62 (9%)	24 (36%)	<0.001
– MI	81 (11%)	53 (8%)	28 (42%)	<0.001
– Heart failure	46 (6%)	28 (4%)	18 (27%)	<0.001
History of diabetes	186 (24%)	163 (23%)	23 (35%)	0.049
History of stroke	79 (10%)	67 (10%)	12 (18%)	0.03
History of peripheral artery disease	56 (7%)	43 (6%)	13 (20%)	<0.001
History of smoking	146 (18%)	136 (19%)	10 (15%)	NS
History of hypertension	678 (88%)	622 (88%)	56 (85%)	NS
History of LVH	82 (11%)	74 (11%)	8 (12%)	NS
History of hypercholesterolaemia	338 (44%)	313/702 (45%)	25/65 (38%)	NS
Three or more risk factors	465 (60%)	407 (53%)	58 (88%)	<0.001
Non-invasive ischaemia testing				
– Abnormal MPS	93/279 (33%)	69/241 (29%)	24/38 (62%)	<0.001
– Abnormal EST	9/328 (3%)	8/302 (3%)	1/26 (4%)	NS
– Abnormal DSE	1/64 (2%)	1/61 (2%)	0/3 (0%)	NS
Coronary angiography	85 (11%)	75 (11%)	10 (15%)	NS
Significant CAD on angiography	19 (2%)	18 (3%)	1 (2%)	NS
Difference in haemoglobin level (mmol/l, mean ± SD)	−2.2 ± 1.0	−2.1 ± 1.0	−2.6 ± 1.0	<0.001

All data are *n* (%) unless stated otherwise

*Tx* transplantation, *MI* myocardial infarction, *LVH* left ventricular hypertrophy, *MPS* myocardial perfusion scintigraphy, *EST* exercise stress testing, *DSE* dobutamine stress echocardiography, *CAD* coronary artery disease

^a^No cardiac event versus cardiac event

## Results

### Baseline characteristics

A total of 770 kidney transplants were performed in 751 patients. There were no missing values for transplant candidates. Mean age at time of transplantation was 54 ± 14 years and the majority were men (64%) (Tab. 1). Almost all patients had at least one risk factor (98%), including hypertension (88%), longer than 1 year on dialysis (49%), hypercholesterolaemia (44%) and age above 60 years (40%). In 58% of transplant candidates at least three risk factors were present. The cardiologist was consulted pre-operatively in 97% of transplant candidates.

### Screening for coronary artery disease

**Fig. 1:**
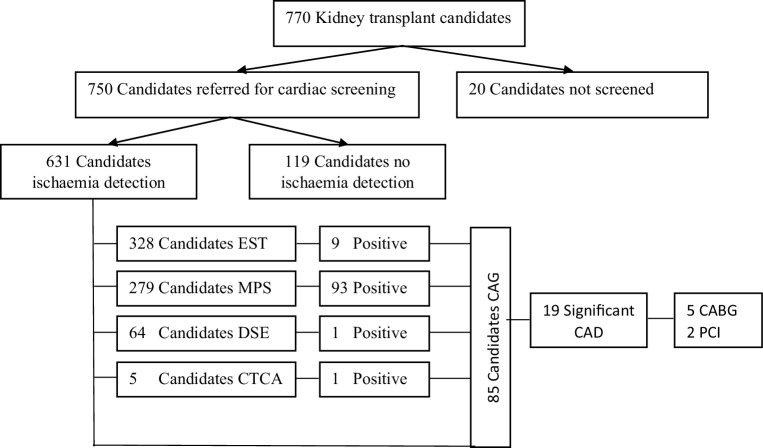
Results of screening for coronary artery disease (*CAD*). *CAG* coronary angiography, *CABG* coronary artery bypass graft, *CTCA* computed tomography coronary angiography, *DSE* dobutamine stress echocardiography, *EST* exercise stress testing, *MPS* myocardial perfusion scintigraphy, *PCI* percutaneous coronary intervention

Screening for significant coronary artery disease (CAD) was performed in 631 transplant candidates (82% of all cases); see Fig. 1. In 546 (87%) transplant candidates only non-invasive ischaemia testing was performed, in 18 (3%) only CAG was performed without any non-invasive ischaemia testing, and in 67 (11%) CAG was performed after non-invasive ischaemia testing. Transplant candidates without testing for CAD were younger (46.6 vs 55.9 years, *p* < 0.001), significantly less often had DM (14% vs 26%, *p* = 0.001), had less often been on dialysis for longer than 1 year (38% vs 51%, *p* = 0.005) and less often had three or more risk factors (46% vs 64%, *p* < 0.001). There was no difference in gender, LVH, hypertension, hypercholesterolaemia or current smoking.

#### Non-invasive testing

Non-invasive ischaemia testing was performed in 613 transplant candidates (80% of all cases). In 63 of those (10%), two non-invasive ischaemia detection modalities were used. The most commonly performed non-invasive test was EST (48.5%), followed by MPS (41.3%). DSE (9.5%) and computed tomography coronary angiography (CTCA) (0.7%) were used in only a low number of cases.

##### 328 ESTs:

47 ESTs were inconclusive (14.3%), 272 were negative for ischaemia (82.9%) and 9 were positive for ischaemia (2.7%). There were 47 inconclusive ESTs, which were followed by 18 MPSs, 6 DSEs and 1 CTCA. Inconclusive ESTs were not followed by adequate testing in 47% (22/47) of transplant candidates. Of those retested, 4 MPSs were positive for reversible ischaemia. Also, 28 MPSs and 3 DSEs were performed in the group with a negative EST. This resulted in another 9 divergent MPSs, 5 with reversible ischaemia and 4 with irreversible ischaemia; no DSE was positive for ischaemia. Three positive ESTs were followed by MPSs, of which only 1 was positive for reversible ischaemia.

##### 279 MPSs:

186 MPSs were negative for ischaemia (66.7%) and 93 MPSs were abnormal (33.3%), 59 showing reversible ischaemia and 34 irreversible ischaemia.

##### 64 DSEs:

Only 1 DSE was positive for ischaemia (1.6%). In 3 negative DSEs, MPS was performed in addition, leading to 1 positive MPS for reversible ischaemia.

##### 5 CTCA:

One CTCA was suspected for significant CAD (20%), the remaining 4 being negative (80%). The suspected CTCA was followed by MPS, which showed no ischaemia.

#### Coronary angiography

CAG was performed in 85 transplant candidates, in 18 transplant candidates without preceding non-invasive ischaemia testing, in 29 transplant candidates after negative ischaemia testing and in 38 transplant candidates after positive ischaemia testing. In 19 transplant candidates there was significant CAD, in 2 without non-invasive ischaemia testing, in 6 with a negative test, and in 11 with a positive test. Positive non-invasive ischaemia testing prior to CAG or three or more risk factors were not predictors for significant CAD, compared to negative or no testing (*p* = 0.18 and *p* = 0.1 respectively). In 7 of 19 transplant candidates with significant CAD, revascularisation was performed electively, 5 times CABG and 2 times PCI. In 3 additional transplant candidates, PCI was performed after approval for kidney transplantation because of an acute coronary syndrome (ACS) while on the waiting list for kidney transplantation. Two of those 3 patients had negative ESTs and 1 patient had reversible ischaemia on MPS but no significant CAD on CAG.

### Primary endpoint

**Fig. 2:**
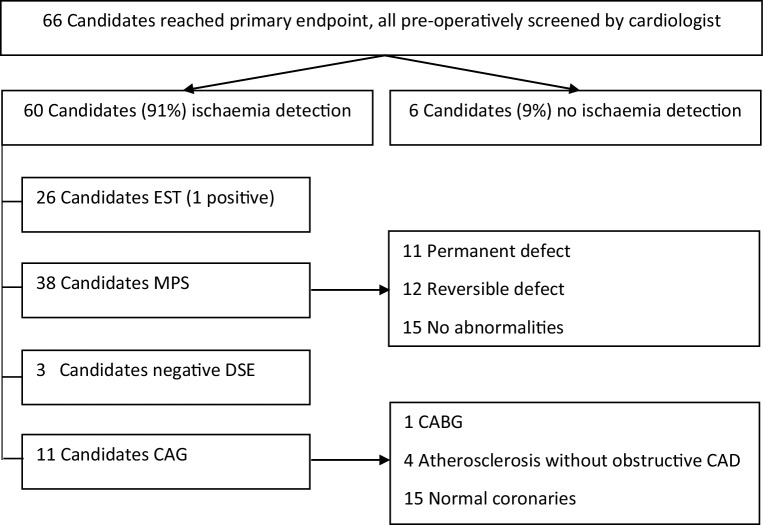
Screening for coronary artery disease (*CAD*) in transplant candidates with a cardiac event after kidney transplantation. *CAG* coronary angiography, *CABG* coronary artery bypass graft, *DSE* dobutamine stress echocardiography, *EST* exercise stress testing, *MPS* myocardial perfusion scintigraphy

The primary endpoint was reached in 66 patients (8.6%, see Fig. 2). There were 4 cardiac deaths; 49 transplant candidates with post-operative ACS, of whom 9 were subsequently revascularised; and 13 transplant candidates with an episode of heart failure requiring admission and medication. All patients reaching the primary endpoint had been consulted by the cardiologist pre-operatively. In 60 patients (91%) screening for CAD was performed. Non-invasive ischaemia detection was performed in 58 patients: 49 patients underwent one test, while 9 patients underwent two tests. There were 24 patients with positive non-invasive ischaemia testing (1 EST and 24 MPSs), of whom 9 were referred for CAG. Two additional patients underwent CAG without non-invasive ischaemia detection. Of the 11 patients that underwent CAG, only 1 had significant CAD (after positive MPS) and was referred for CABG. Sensitivity for MPS was 77% and specificity 41%, and for EST 33% and 75% respectively.

Of all transplant candidates with non-invasive ischaemia testing, 103 were positive for ischaemia (16.8%). Transplant candidates with a positive non-invasive ischaemia test had significantly more events than patients with a negative test (24% vs 6%, *p* < 0.001) or no non-invasive ischaemia test (24% vs 5%, *p* < 0.001). When the non-invasive ischaemia tests were analysed separately, only transplant candidates with a divergent MPS, but not DSE, CTCA or EST, had significantly more events than transplant candidates with normal or no MPS (26% vs 8%, *p* < 0.001 and 26% vs 6%, *p* < 0.001).

Tab. 1 shows that patients that reached the primary endpoint more often received a deceased-donor kidney transplant (*p* = 0.001), more often had a cardiovascular history (59% vs 14%, *p* < 0.001), more often had DM (35% vs 23%, *p* = 0.049), were more often older than 60 years (68% vs 38%, *p* < 0.001), and were more often on dialysis longer than 1 year (65% vs 47%, *p* = 0.004). Also they more often had three or more clinical risk factors (88% vs 53%,* p* < 0.001), more often had delayed graft function (38% vs 21%, *p* = 0.001), more often had a larger decrease in haemoglobin post-operatively (−2.1 ± 1 vs −2.6 ± 1, *p* < 0.001) and more often had abnormal MPS (62% vs 29%, *p* < 0.001). There were no differences in gender, smoking habit, LVH, hypertension, hypercholesterolaemia, positive EST, DSE or CTCA.

In multivariable analysis, history of myocardial infarction (*p* < 0.001), history of heart failure (*p* < 0.001), age at transplantation (*p* = 0.005), difference in haemoglobin level (*p* = 0.003) and any positive non-invasive ischaemia testing (*p* = 0.01) remained as significant predictors of the study endpoint (Tab. 2). Analysis of the influence of MPS separately failed to reach significance in multivariable analysis (*p* = 0.08).

**Table 2:** Multivariate analysis

Variable	RR	95% confidence interval	*p*
Age at transplant (per year increase)	1.04	1.0–1.1	0.005
History of MI	4.3	2.3–8.3	<0.001
History of heart failure	5.4	2.5–11.4	<0.001
Positive non-invasive ischaemia testing	2.6	1.4–5.1	0.01
Haemoglobin difference (per mmol decrease)	1.5	1.2–2.0	0.003

*MI* myocardial infarction, *RR* relative risk

## Discussion

The main finding of this study is that in 8.6% of renal transplant patients, there was a cardiovascular event within 3 months after transplantation. These events occurred despite pre-transplant cardiac evaluation in almost all patients and screening for significant CAD in 82% of patients and the incidence is higher than the predicted 1–5% (intermediate) risk for 30-day mortality or myocardial infarction in the European Society of Cardiology (ESC) guideline for non-cardiac surgery. Of the patients that reached the study endpoint, 60% had negative pre-operative non-invasive ischaemia testing and were subsequently accepted for renal transplantation. Sensitivity for MPS and EST was 77% and 33% respectively, while specificity was 41% and 75% respectively. DSE was not performed in enough transplant candidates to reliably determine sensitivity and specificity. Non-invasive tests for CAD have a lower sensitivity and specificity in patients with renal failure than in the general population. In populations with chronic kidney disease stage 5, DSE and MPS had sensitivities varying from 0.44 to 0.89 and 0.29 to 0.92 and specificities ranging from 0.71 to 0.94 and 0.67 to 0.89, respectively, for identifying 1 or more coronary stenoses >70% [15]. The reason for the decreased sensitivity and specificity in the population with ESRD might be that the target heart rate has not been attained because of a bad physical condition, anaemia or the use of beta-blockers. Although positive non-invasive ischaemia tests in ESRD patients show poor correlation with significant CAD, both positive DSE and MPS have been associated with cardiac death and myocardial infarction, both while on the waiting list and post-transplantation [16, 17]. We also found that, of all four non-invasive ischaemia tests, MPS was most prevalent in the population with a cardiac event post-transplantation. However, in multivariable analysis, no single non-invasive ischaemia detection modality was able to predict cardiovascular events after kidney transplantation. When analysed together, any positive non-ischaemia detection test was correlated with a post-transplant cardiovascular event. This could be explained by the inconsistency in the tests used and the use of tests that are non-discriminatory in this population.

Non-invasive ischaemia testing was the only predictor of a cardiovascular event that can be influenced by the cardiologist, the others being age and history of myocardial infarction or heart failure. Therefore, it seems important not only to use uniform testing but also a better test. Very recently, Winther and co-workers showed that in 154 patients with ESRD, evaluated for kidney transplantation, MPS was not a predictor of major adverse cardiovascular events (MACE) or death [18]. However, they showed for the first time that CTCA significantly predicted MACE and death. Furthermore, they were able to identify a group with a low risk of MACE and mortality, namely patients with less than three risk factors and a coronary artery calcium score (CACS) of less than 400. Based on their findings they propose an interesting new algorithm of a combination of risk factors and CACS and subsequent CTCA for cardiac screening in renal transplant candidates.

In order to reduce cardiovascular complications peri-operatively, patients with significant CAD should be adequately treated. In our study, of 103 positive non-invasive ischaemia tests only 35 were followed by CAG (6 positive ESTs, 24 MPSs with reversible ischaemia, 5 MPSs with irreversible ischaemia). Most pre-transplant cardiac evaluations are performed in the referring hospital, so we do not know why CAG was performed in such a low number of cases after positive ischaemia detection. On CAG, 19 patients had significant CAD (22%), of whom 7 were revascularised and 12 were managed medically. Eventually, 10 patients (only 1.3% of all transplant candidates) were revascularised, as 3 additional patients experienced an ACS while on the waiting list and were revascularised. There was no difference in primary endpoint between medically managed or revascularised patients, but the numbers are low and of course patients are not randomised, so probably the selection of worse patients was treated.

Routine prophylactic revascularisation before low- and intermediate-risk surgery in patients with proven CAD is not recommended in the ESC guideline on cardiovascular assessment and management for non-cardiac surgery (class III, level of evidence B). This recommendation is based upon the CARP study [19], in which patients with significant CAD that were scheduled for elective major vascular surgery were randomised to pre-operative revascularisation or no revascularisation. Pre-operative revascularisation did not alter long-term outcome. The 1992 study by Manske and co-workers is the only randomised controlled trial that evaluated coronary revascularisation in renal transplant candidates [20]. A total of 26 asymptomatic insulin-dependent diabetic patients were randomised to revascularisation or medical treatment. Ten of 13 medically managed and 2 of 13 revascularised patients had a cardiovascular endpoint with a median follow up of 8.4 months. Manske et al. concluded that diabetic renal transplant candidates should be screened for silent CAD, as coronary revascularisation might decrease cardiac morbidity and mortality. These results must be interpreted with caution as medically treated patients were only treated with a calcium channel blocking drug and acetylsalicylic acid, which is nowadays thought to be inadequate. Since then, several observational studies have been published with mixed results. In a study by de Lima et al., 44% of 519 pre-transplant patients had significant CAD on CAG. Most patients were treated conservatively, while only 13% had a cardiac intervention (at the discretion of the attending cardiologist). No difference was found in cardiac-event-free and patient survival between the patients with and without an intervention [21]. In a later study, de Lima et al. showed that patients with significant CAD (>70% stenosis) experienced more coronary events compared to patients with less significant lesions. There was no difference in mortality or events between patients treated medically or by intervention, although severity of CAD in the latter was higher [22]. In contrast, in a retrospective analysis, Kahn et al. showed that patients with medically managed obstructive CAD had significantly higher rates of death at 5 years post-transplantation when compared to those who were revascularised before transplantation [23]. In a pre-kidney transplant population in London, Kumar et al. showed excellent survival rates and low complication rates after revascularisation with an aggressive intervention protocol. Renal transplant candidates demonstrated 1‑ and 3‑year survival rates of 98.0% and 88.4% in those who underwent revascularisation and then transplantation, 75.0% and 37.1% in those who did not undergo revascularisation or transplantation, and 94.0% and 90.0% in those who underwent revascularisation and remained on a transplant list [24]. Overall, cardiovascular intervention before renal transplantation has not unanimously been shown to reduce the prevalence of cardiovascular disease (CVD) after transplantation. However, these results must be considered with caution because, by definition, patients referred for intervention had more serious and widespread disease than those on medical treatment because referral was at the discretion of the local cardiologist. This means that the CVD-free survival of the population on medical treatment should have been far better because they were a selection of patients with less severe disease. Nonetheless, at best, the results were comparable or even worse.

The current study has some limitations. First, this is a retrospective study and therefore only an association and no causation can be extracted from the results. Secondly, many patients were screened by cardiologists from referring hospitals and we had access only to the correspondence from the cardiologist. Therefore, we did not judge the results of non-invasive ischaemia testing and CAG ourselves. Thirdly, the cohort under study dates from 2010 to 2013, but we believe that the results are still valid as ischaemia detection methods have not changed since then and cardiovascular events post-transplantation have not decreased.

## Conclusion

In conclusion, in this retrospective study we showed that the risk of experiencing a myocardial event after kidney transplantation is high for an elective procedure and higher than the intermediate risk according to the ESC guidelines. This risk was predicted by age, pre-transplant myocardial infarction or heart failure, post-operative decrease in haemoglobin and positive non-invasive ischaemia testing. There was a trend for MPS to predict cardiovascular events post-transplantation, while CAG did not. However, the CAG was performed in only a low number of cases. Pre-transplant revascularisation did not prevent cardiovascular events post-transplantation, although again the numbers are very low. These results are in line with those of earlier studies and show the complexity of cardiac screening in renal transplant candidates. A prospective randomised controlled study to unravel the indications, methods and consequences of ischaemia detection (i.e. with or without revascularisation) could provide insight into how to prevent cardiovascular events post-transplantation.
